# Evolution of Apparent Diffusion Coefficient and Fractional Anisotropy in the Cerebrum of Asphyxiated Newborns Treated with Hypothermia over the First Month of Life

**DOI:** 10.1155/2015/653727

**Published:** 2015-07-02

**Authors:** Saskia Kwan, Elodie Boudes, Anouk Benseler, Guillaume Gilbert, Christine Saint-Martin, Michael Shevell, Pia Wintermark

**Affiliations:** ^1^Division of Newborn Medicine, Department of Pediatrics, Montreal Children's Hospital, McGill University, Montreal, QC, Canada H3H 1P3; ^2^MR Clinical Science, Philips Healthcare, Montreal, QC, Canada H2S 2J3; ^3^Department of Pediatric Radiology, Montreal Children's Hospital, McGill University, Montreal, QC, Canada H3H 1P3; ^4^Division of Pediatric Neurology, Department of Pediatrics, Montreal Children's Hospital, McGill University, Montreal, QC, Canada H3H 1P3

## Abstract

The objective of this study was to assess the evolution of diffusion-weighted imaging (DWI) and diffusion-tensor imaging (DTI) over the first month of life in asphyxiated newborns treated with hypothermia and to compare it with that of healthy newborns. Asphyxiated newborns treated with hypothermia were enrolled prospectively; and the presence and extent of brain injury were scored on each MRI. Apparent diffusion coefficient (ADC) and fractional anisotropy (FA) values were measured in the basal ganglia, in the white matter and in the cortical grey matter. Sixty-one asphyxiated newborns treated with hypothermia had a total of 126 ADC and FA maps. Asphyxiated newborns developing brain injury eventually had significantly decreased ADC values on days 2-3 of life and decreased FA values around day 10 and 1 month of life compared with those not developing brain injury. Despite hypothermia treatment, asphyxiated newborns may develop brain injury that still can be detected with advanced neuroimaging techniques such as DWI and DTI as early as days 2-3 of life. A study of ADC and FA values over time may aid in the understanding of how brain injury develops in these newborns despite hypothermia treatment.

## 1. Introduction

Birth asphyxia and its resulting neonatal encephalopathy are associated with significant mortality and long-term neurodevelopmental complications, such as cerebral palsy, intellectual disability, learning difficulties, and other cognitive deficits. Therapeutic hypothermia has become the standard of care for these asphyxiated newborns to possibly prevent the development of brain injury and improve their long-term outcome [1–3].

Magnetic resonance imaging (MRI) is the standard imaging technique to define the presence and extent of brain injury in these newborns and to provide a prognosis [4, 5]. Before the cooling era, the evolution over time of diffusion-weighted imaging (DWI) [6–9] and diffusion-tensor imaging (DTI) [10, 11] had been widely studied to understand the development of brain injury in asphyxiated newborns [12–14]. Abnormalities of diffusion were typically most evident within the first few days of life and then were “pseudonormalized” over the first two weeks of life; structural abnormalities became clearly visible on conventional imaging by days 7–10 of life [15–17]. These abnormal changes within the first few days of life [18–20] have been repeatedly confirmed as objective measures of prognosis [21–23], often before conventional imaging changes are clearly notable [24–26].

Similar studies are currently lacking for asphyxiated newborns treated with hypothermia, and some have postulated that hypothermia treatment may modify the evolution of these sequences over time [27–31]. Literature on the brain imaging of asphyxiated newborns treated with hypothermia typically uses the brain imaging performed after the completion of the hypothermia [27–29, 31, 32]. Most available studies mainly address the incidence and type of brain injury observed following hypothermia treatment and their predictive value for subsequent neurological impairment [4, 5, 32]. Only a few studies have begun to investigate the value of diffusion parameters for these newborns [27–29], mostly at one time-point [28, 31, 33, 34]. However, none of these studies longitudinally follows the newborns so as to accurately define the evolution of these diffusion parameters within the first days of life and over the first month of life so as to understand better how the brain injuries develop in these newborns despite hypothermia treatment.

Thus, this present study was designed to compare the evolution of DWI and DTI within the first days of life and over the first month of life in asphyxiated newborns treated with hypothermia and to compare it with that of healthy newborns. We hypothesize that ADC values may already be abnormal in the injured brain areas within the first days of life in these asphyxiated newborns even if they are treated with hypothermia and that FA values may help us understand better the later development of brain injury despite hypothermia treatment.

## 2. Materials and Methods

### 2.1. Patients

We conducted a prospective cohort study of term asphyxiated newborns admitted to our neonatal intensive care unit from 2010 to 2014, who met established criteria for induced hypothermia [1–3]: (1) gestational age ≥ 36 weeks and birth weight ≥ 1800 g, (2) evidence of fetal distress, for example, a history of acute perinatal event, cord pH ≤ 7.0, or base deficit ≤ −16 mEq/L, (3) evidence of neonatal distress, such as an Apgar score ≤ 5 at 10 minutes, postnatal blood gas pH obtained within the first hour of life ≤ 7.0 or base deficit ≤ −16 mEq/L, or continued need for ventilation initiated at birth and continued for at least 10 minutes, and (4) evidence of moderate or severe encephalopathy obtained by standardized neurological exam and/or amplitude-integrated electroencephalogram. Eligible patients received whole-body cooling to an esophageal temperature of 33.5°C, initiated by 6 hours of life and continued for 72 hours (unless contraindications developed), and were then slowly rewarmed. Four additional healthy term newborns were also enrolled as controls. The research protocol was approved by the Institutional Review Board and informed parental consent was obtained in all cases.

### 2.2. Brain Magnetic Resonance Imaging (MRI)

As per standard clinical protocol at our institution, a brain magnetic resonance imaging (MRI) scan was performed for all these newborns around day 10 of life. When possible (i.e., when the parents consented for their newborns to have additional MRIs, when the newborns were hemodynamically stable, and when a team of a nurse and a respiratory therapist was available to go to the MRI), newborns were enrolled in an MRI research study, and MRI scans were performed on day 1 of life, on days 2-3 of life, around day 10 of life, and around 1 month of life. These time-points were chosen to ensure the absence of antenatal brain injury (day 1 of life), to assess early patterns of injury (days 2-3 of life), and to define the extent of later definitive structural brain injuries (around day 10 of life and around 1 month of life). Patients receiving hypothermia had their therapy maintained during the MRI scan without any adverse events [35]. Any ventilation, inotropic support, or sedation was maintained during the MRI scanning process, and additional sedation was avoided. Motion artifacts were minimized by wrapping the neonates in an MR imaging-compatible vacuum cushion. Each of the four healthy newborns was scanned at the four different time-points: on day 1 of life, on days 2-3 of life, around day 10 of life, and around 1 month of life.

The magnetic resonance imaging (MRI) scans were performed using a 3T clinical system (Achieva X, Philips Healthcare, Best, Netherlands). Each MRI research study included a 3D T1-weighted gradient-echo (TR/TE, 24/4.6 ms; matrix size, 180 × 180; FOV, 180 mm; flip angle, 30 degrees; 104 sagittal slices, with a section thickness of 1.0 mm and multiplanar reformations in axial and coronal planes), a high resolution turbo spin echo T2-weighted imaging (TSE) (TR/TE, 5000/90 ms; TSE factor, 15; matrix size, 300 × 300; FOV, 150 mm; flip angle, 90 degrees; 27 axial slices, with a section thickness of 3.0 mm), and a single shot echo planar imaging (EPI) diffusion-tensor imaging (DTI) sequence with isotropic resolution (TR/TE, 5937.8/69 ms; matrix size, 100 × 100; FOV, 180 mm; SENSE factor 2; 32 directions; *b* values, 0 and 750 s/mm^2^; 46 axial slices, with a section thickness of 1.8 mm).

Pediatric neuroradiologists, who were blind to the clinical conditions of the infants, interpreted the MRI studies of the asphyxiated newborns treated with hypothermia and the healthy newborns. They reported the presence and extent of brain injury in the cerebrum as per the previously described magnetic resonance imaging scoring system [36]. The MRI scores obtained around day 10 of life were used as the reference to determine the extent of the brain injury for each patient [17, 37–39].

**Figure 1:**
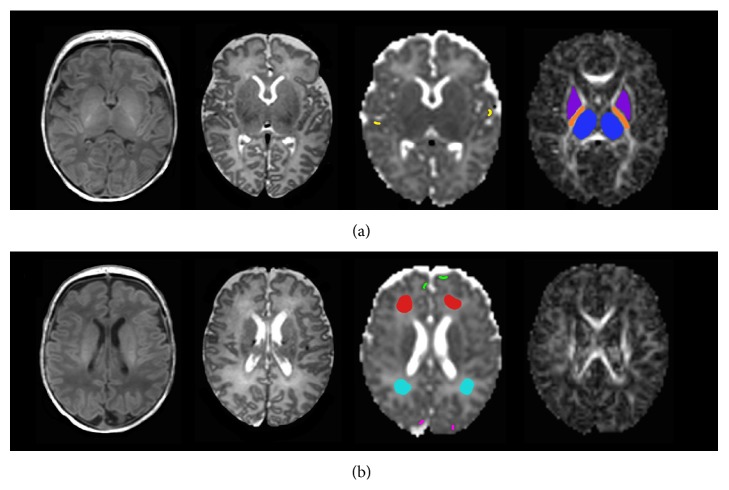
Regions of interest. Axial T1-weighted images, T2-weighted images, apparent diffusion coefficient (ADC) maps, and fractional anisotropy (FA) maps at two different levels of cerebrum showing the manually drawn regions of interest: (a) lower level and (b) higher level. ADC and FA values were measured in eight different regions of interest in the cerebrum: that is, in the thalamus, the posterior limb of the internal capsule and the lentiform nucleus, the white matter (within the frontal and posterior white matter), and the cortical grey matter (within the frontal, parietal, and occipital cortices). Regions of interest were always drawn by the same observer by looking at the same time at the ADC and FA maps and the corresponding T1- and T2-weighted imaging.

In addition, the Brain Extraction Tool (Oxford Centre for Functional Magnetic Resonance of the Brain, Oxford University, Oxford, UK) was used to remove all extracerebral tissues (i.e., eyes, meninges, and skull) from the images [40]. Then, apparent diffusion coefficient (ADC) and fractional anisotropy (FA) maps were generated for the whole brain using the tools of the Diffusion Imaging in Python (DIPY) package (Sherbrooke Connectivity Imaging Lab, Sherbrooke University, Quebec, Canada) [41]. To assess the evolution of ADC and FA during the first month of life in asphyxiated newborns treated with hypothermia, ADC and FA values were measured in eight different regions of interest in the cerebrum (Figure 1): regions of interest in the thalamus, the posterior limb of the internal capsule and the lentiform nucleus, the white matter (within frontal and posterior white matter), and the cortical grey matter (within the frontal, parietal, and occipital cortices), as described in other studies [42]. The regions of interest always were drawn by the same observer on the axial ADC and FA maps using ImageJ (Image Processing and Analysis in Java) [43]. To accurately identify the regions of interest in the cerebrum, axial T1- and T2-weighted imaging was used in conjunction with the axial ADC and FA maps. Measurements were obtained in the right and left sides in these different regions of interest and then averaged. The measurements were repeated twice for each region of interest to increase the robustness of repeated measures.

### 2.3. Statistical Analysis

For the analysis of ADC and FA measurements in each region of interest, asphyxiated newborns treated with hypothermia were categorized into two subgroups based on the presence or absence of brain injury in that region of interest on their conventional MRI performed around day 10 of life. Statistical analysis was performed according to the day of life when the MRI scan was taken. For each region of interest, ADC and FA values were compared between the three different groups (i.e., healthy newborns, asphyxiated newborns treated with hypothermia who did not develop later brain injury, and asphyxiated newborns treated with hypothermia who developed later brain injury) using Mann-Whitney *U* tests. A *p* value < 0.05 was considered a priori as statistically significant. All statistical analyses were performed using GraphPad Prism (GraphPad Software Inc., San Diego, CA, USA).

## 3. Results

**Figure 2:**
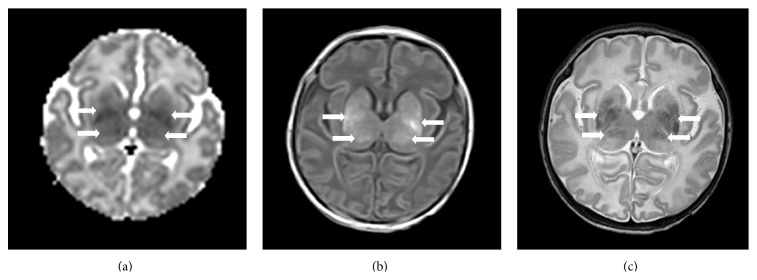
Brain MRIs of a term asphyxiated newborn treated with hypothermia, performed on day 2 of life (a) and on day 9 of life (b-c). (a) The apparent diffusion coefficient map on day 2 of life for this patient shows a restricted diffusion involving the thalami and lentiform nuclei (arrows). (b and c) The T1-weighted imaging (b) and T2-weighted imaging (c) confirm the injury within the thalami and lentiform nuclei (arrows).

Sixty-one asphyxiated newborns treated with hypothermia were included in the present study (Table 1). Forty-nine percent (30/61) did not develop later brain injury, and 51% (31/61) developed later brain injury. Twenty-two percent (7/31) developed basal ganglia injury (Figure 2), 22% (7/31) developed watershed injury, and 56% (17/31) developed near-total injury.

A total of 126 MRI scans were performed on these 61 asphyxiated newborns treated with hypothermia. Thirteen were obtained on day 1 of life, 32 on days 2-3 of life, 54 around day 10 of life (mean: day 11 of life; range: days 7–15 of life), and 27 around one month of life (mean: day 33 of life; range: days 29–45 of life). Eleven newborns were scanned at the four different time-points; 9 were scanned only at three time-points, 14 only at two time-points, and 27 only at one time-point. Three of the enrolled newborns died during the first week of life; for these patients, the autopsy results confirmed the brain injury observed on the early scans during hypothermia treatment.

In the asphyxiated newborns treated with hypothermia who developed or did not develop later brain injury, the ADC values (Table 2) were significantly decreased on day 1 of life and on days 2-3 of life in the different regions of interest (thalamus, posterior limb of internal capsule, lentiform nucleus, and anterior white matter, as well as frontal cortical grey matter) when compared with the healthy newborns; the same trend was observed in the posterior white matter, but it did not reach statistical significance. In addition, in the asphyxiated newborns developing brain injury, the ADC values were significantly decreased on days 2-3 of life in the different regions of interest, that is, the thalamus (*p* = 0.01), the posterior limb of the internal capsule (*p* = 0.004), the lentiform nucleus (*p* = 0.0002), the anterior white matter (*p* = 0.02), the posterior white matter (*p* = 0.02), and the parietal cortical grey matter (*p* = 0.04), when compared with those without brain injury. The ADC values on day 1 of life were significantly decreased only in the thalamus (*p* = 0.01) and in the lentiform nucleus (*p* = 0.04) in the newborns developing later brain injury, compared with those without brain injury. Around day 10 of life, the ADC values were significantly increased only in the thalamus and in the frontal cortical grey matter in the asphyxiated newborns who developed brain injury when compared with those without brain injury; however, this was not anymore the case around day 30 of life. The ADC values in the posterior white matter were significantly increased around day 10 of life and around day 30 of life in the asphyxiated newborns treated with hypothermia developing or not developing brain injury when compared with the healthy newborns. This result was also observed around day 30 of life in the parietal cortical grey matter and the anterior white matter.

FA values (Table 3) were not different between the three groups on day 1 of life and on days 2-3 of life in the different regions of interest. In the asphyxiated newborns treated with hypothermia who developed later brain injury, the FA values were decreased around day 10 of life and around day 30 of life in the thalamus (*p* = 0.02 around day 10 of life and *p* = 0.002 around day 30 of life), the posterior limb of internal capsule (*p* < 0.0001 around day 10 of life and around day 30 of life), the lentiform nucleus (*p* = 0.0004 around day 10 of life), and the anterior white matter (*p* = 0.03 around day 10 of life and *p* = 0.01 around day 30 of life) when compared with those infants without brain injury and the healthy newborns. The FA values in the posterior white matter were not different between the asphyxiated newborns developing or not developing brain injury, but they were significantly reduced when comparing these newborns to the healthy newborns. The FA values also decreased in the lentiform nucleus around day 10 of life (*p* = 0.009) and in the anterior white matter on day 30 of life (*p* = 0.07) in the asphyxiated newborns not developing brain injury, compared with the healthy newborns. The FA values in the cortical grey matter were not different between the three groups around day 10 of life and around day 30 of life.

## 4. Discussion

Hypothermia treatment has been associated with improved brain metabolism and preserved brain microstructure [28] and has been postulated to slow down the evolution of diffusion changes in asphyxiated newborns [27–31]. Up to now, most studies that have investigated the value of diffusion parameters for these newborns only use imaging performed at one time-point [28, 31, 33, 34]. Massaro et al. [34] did not show any ADC difference between the newborns developing or not developing brain injury in 40 asphyxiated newborns treated with hypothermia on MRI scanned around day 7 of life (range: days 5–12 of life, eight newborns developed brain injury). Bonifacio et al. [28] showed that ADC values were higher and closer to normal in 29 asphyxiated newborns treated with hypothermia scanned around day 5 of life (range: days 4–6 of life, 12 newborns developed brain injury), compared with the asphyxiated newborns, who were not treated [28]; however, the newborns treated with hypothermia were usually imaged one day later compared with the not treated newborns, and “pseudonormalization” may have accounted for some of these reduced diffusion abnormalities [28]. Thakur et al. [31] concluded that ADC values are more likely to be abnormal when they are performed closer to the cessation of hypothermic treatment between days 4 and 8 of life; however, they did not perform any imaging before day 4 of life in their study of 44 asphyxiated newborns treated with hypothermia scanned around day 7 of life (range: days 4–17 of life, 14 newborns developed later brain injury) [31]. Lee et al. [33] demonstrated that FA values were lower in the injured brain areas in 36 asphyxiated newborns treated with hypothermia scanned around day 9 of life (range: days 5–18 of life, 24 newborns developed brain injury) [33]. Only two previous studies have investigated the diffusions changes on serial imaging [27, 29]. Gano et al. [29] suggested that hypothermia seemed to attenuate the severity and progression of brain injury; however, they studied only six asphyxiated newborns treated with hypothermia scanned on days 1, 3, and 10 of life, and only 3 newborns developed brain injury [29]. Bednarek et al. [27] concluded that therapeutic hypothermia may “slow down” the evolution of the mean diffusivity changes, with a delay in “pseudonormalization” occurring after the tenth day of life in 23 asphyxiated newborns treated with hypothermia with brain injury on MRI performed around day 6 of life (range: days 0–17 of life, 12 had serial imaging) compared with days 6–8 of life in the control group not treated with hypothermia [27].

The present study presents the evolution of ADC and FA values over the first month of life of 61 asphyxiated newborns treated with hypothermia (31 newborns developed brain injury) and compares them to the values obtained in four healthy newborns. In asphyxiated newborns treated with hypothermia who developed later brain injury, ADC values typically started to decrease on day 1 of life, were significantly decreased on days 2-3 of life, and then were “pseudonormalized,” compared with the asphyxiated newborns without brain injury; FA values were not different on days 1–3 of life but decreased around day 10 and 1 month of life when compared with the same newborns. Advanced neuroimaging techniques such as DWI and DTI still enable the detection of changes related to the later development of brain injury in asphyxiated newborns treated with hypothermia, as early as days 2-3 of life. This result is similar to what was described regarding normothermic asphyxiated newborns [6–15]. The early decrease of ADC within the first days of life was still visible in our cohort treated with hypothermia, as previously suggested by other authors [27].

Interestingly, ADC values were significantly decreased on day 1 of life and on days 2-3 of life for all the different regions of interest in all the asphyxiated newborns treated with hypothermia compared with the healthy newborns. Hypothermia itself has been previously described as modifying diffusion values such as ADC measurements [27, 44]. ADC values are known to have a dependence on temperature (2.4% per 1°C change) [27, 44] and thus diffusion measurements made during hypothermia treatment (i.e., at an esophageal temperature of 33.5°C) are expected to be lower than those that would be made at normal body temperature.

Intriguingly, FA values were different in the posterior white matter around day 10 of life and around day 30 of life for the asphyxiated newborns treated with hypothermia who did not develop brain injury compared with the healthy newborns; a similar trend was observed in the lentiform nucleus around day 10 of life and in the anterior white matter on day 30 of life. Interestingly, when looking more carefully at this analysis, a cluster of six newborns with no reported brain injury in these regions of interest had decreased FA values compared with the healthy newborns, but similar FA values compared with the asphyxiated newborns presenting injury in these regions of interest. For these six newborns, the ADC values on days 2-3 of life were not different compared with the healthy newborns, which suggests that no acute sign of brain injury existed in those regions of interest. However, all these patients had brain injury in the thalamus and the posterior limb of internal capsule. All together, these results suggest that injury in these regions of interest (anterior and posterior white matter and lentiform nucleus) may be indirect by way of transneuronal degeneration related to damage in other interconnected areas of the brain [13, 45, 46]. These results suggest that FA measurements may enable the detection of subtle white matter injuries that are otherwise not visible on conventional imaging.

FA values were not different in the cortical grey matter at the different time-points between asphyxiated newborns developing or not developing brain injury, even if ADC values tended to be lower within the first days of life in these regions of interest. The small number of newborns with brain injury in the regions of interest within cortical grey matter probably partly explains the lack of statistical significance in these regions of interest. Also it was technically challenging to always recognize the same specific regions in the cortical grey matter from one patient to the other and to avoid volume averaging with white matter when measuring in the cortical ribbon. The studied cortical gray matter areas may have been “contaminated” to varying degrees by subarachnoid fluid or by subcortical white matter. It is probably why most other studies measuring ADC and FA have focused on measuring these parameters only in the white matter and in the basal ganglia [13, 20, 29, 33, 47], since these regions of interest are more easily identifiable and provide reproducible and reliable identifiable measurements from one patient to the other.

The strength of the current study is the serial MRI design in the same newborns to follow the evolution of their brain injuries over time in a large number of asphyxiated newborns treated with hypothermia. When possible, imaging was repeated on day 1 of life, on days 2-3 of life, around day 10 of life, and/or around 1 month of life; these time-points were chosen to ensure the absence of antenatal brain injury (day 1 of life), to assess early patterns of injury (days 2-3 of life), and to define the extent of definitive brain injuries (around day 10 of life and around 1 month of life). In this study, the newborns were not imaged between days 4 and 6 of life, considering the possible risk of not seeing the full extent of the brain injury [18, 34, 39]. A group of healthy newborns scanned at the four different time-points were also included for comparison but only included four newborns. As the sample size was limited, we thus did not test whether the ADC and FA values were predictive or not of late injury. Also, it would have been ideal to study a group of asphyxiated newborns not treated with hypothermia so as to determine how the hypothermia treatment influenced the magnitude of the severity of the observed changes. However, since cooling is now the standard of care, it is no longer ethically possible to not receive hypothermia to assess the evolution over time of DWI and DTI. In addition, neurodevelopmental studies of these newborns also would be recommended to better understand how these injuries unfold into eventual neurodevelopmental impairments. Additional studies measuring the three principal eigenvectors that can describe diffusion in and around a lesion may provide additional insight into injury evolution and the response of the brain to the insult [48]. An atlas-based DTI analysis in which these results would be compared with healthy controls would also enable a better understanding of these injuries.

In conclusion, advanced neuroimaging techniques such as DWI and DTI still enable the detection of changes related to the development of later brain injury of asphyxiated newborns treated with hypothermia, as early as days 2-3 of life, similarly to what was described for normothermic asphyxiated newborns. In addition, repeated FA measurements may enable the detection of subtle white matter injuries that are otherwise not visible on conventional imaging. Further studies with serial MRI design focusing on a larger group of asphyxiated newborns treated with hypothermia who develop brain injury are needed to further understand how brain injuries develop in these newborns despite hypothermia treatment. These studies may improve the accuracy of prognostic information given to the parents of such infants.

## Figures and Tables

**Table 1:** Clinical characteristics of the asphyxiated newborns treated with hypothermia.

Variables	All patients (*n* = 61)
Gestational age (weeks), mean ± SD	39.21 ± 1.52
Birth weight (g), mean ± SD	3402 ± 703
Sex	
Male, *n* (%)	34 (56)
Female, *n* (%)	27 (44)
Arterial cord pH, mean ± SD	6.98 ± 0.20
Initial postnatal blood gas pH, mean ± SD	7.04 ± 0.16
Initial modified Sarnat score	
Score 1 (mild)	10 (16)
Score 2 (moderate)	41 (67)
Score 3 (severe)	10 (16)
Initiation of hypothermia (hours), mean ± SD	5.22 ± 1.30

The ten newborns with initial modified Sarnat score of 1 were cooled because their initial background pattern on amplitude-integrated electroencephalogram showed moderate encephalopathy.

**Table 2:** Comparison of apparent diffusion coefficient (ADC) values (×10^−3^ mm^2^/s) in each region of interest between healthy newborns and asphyxiated newborns treated with hypothermia developing or not brain injury, on day 1 of life, on days 2-3 of life, around day 10 of life, and around day 30 of life.

	Thalamus	Posterior limb of internal capsule	Lentiform nucleus	Anterior white matter	Posterior white matter	Frontal cortical grey matter	Parietal cortical grey matter	Occipital cortical grey matter
On day 1 of life								
Healthy newborns	1.076 ± 0.031 (*n* = 4)	1.085 ± 0.041 (*n* = 4)	1.144 ± 0.016 (*n* = 4)	1.760 ± 0.086 (*n* = 4)	1.494 ± 0.135 (*n* = 4)	1.208 ± 0.096 (*n* = 4)	1.128 ± 0.055 (*n* = 4)	1.080 ± 0.084 (*n* = 4)
Asphyxiated newborns—no injury	1.014 ± 0.044 (*n* = 6)	1.018 ± 0.052 (*n* = 6)	1.096 ± 0.056 (*n* = 8)	1.634 ± 0.113 (*n* = 12)	1.444 ± 0.085 (*n* = 12)	1.042 ± 0.050 (*n* = 13)	1.005 ± 0.053 (*n* = 8)	0.959 ± 0.053 (*n* = 13)
Asphyxiated newborns—injury	0.891 ± 0.179 (*n* = 7)	0.960 ± 0.137 (*n* = 7)	1.008 ± 0.130 (*n* = 5)	1.522 ± 0.114 (*n* = 1)	1.451 ± 0.036 (*n* = 1)		0.936 ± 0.062 (*n* = 5)	
*p* value: no injury versus healthy	0.006^*∗∗*^	0.012^*∗*^	0.025^*∗*^	0.008^*∗∗*^	ns	0.0005^*∗∗∗*^	0.0002^*∗∗∗*^	0.002^*∗∗*^
*p* value: injury versus healthy	0.0003^*∗∗∗*^	0.0006^*∗*^	0.0002^*∗∗∗*^	—	—	—	0.004^*∗∗*^	—
*p* value: injury versus no injury	0.010^*∗*^	ns	0.042^*∗*^	—	—	—	ns (0.069)	—

On days 2-3 of life								
Healthy newborns	1.075 ± 0.036 (*n* = 4)	1.111 ± 0.029 (*n* = 4)	1.177 ± 0.027 (*n* = 4)	1.709 ± 0.147 (*n* = 4)	1.477 ± 0.115 (*n* = 4)	1.210 ± 0.085 (*n* = 4)	1.138 ± 0.056 (*n* = 4)	1.124 ± 0.059 (*n* = 4)
Asphyxiated newborns—no injury	1.019 ± 0.058 (*n* = 15)	1.033 ± 0.058 (*n* = 17)	1.109 ± 0.063 (*n* = 20)	1.558 ± 0.123 (*n* = 29)	1.448 ± 0.121 (*n* = 27)	1.084 ± 0.074 (*n* = 31)	1.026 ± 0.051 (*n* = 21)	1.030 ± 0.078 (*n* = 29)
Asphyxiated newborns—injury	0.891 ± 0.204 (*n* = 17)	0.925 ± 0.144 (*n* = 15)	0.975 ± 0.175 (*n* = 12)	1.447 ± 0.121 (*n* = 3)	1.312 ± 0.171 (*n* = 5)	1.030 ± 0.082 (*n* = 1)	0.963 ± 0.102 (*n* = 11)	1.002 ± 0.073 (*n* = 3)
*p* value: no injury versus healthy	0.016^*∗*^	0.002^*∗∗*^	0.005^*∗∗*^	0.018^*∗*^	ns	0.001^*∗∗*^	0.0002^*∗∗∗*^	0.001^*∗∗*^
*p* value: injury versus healthy	0.003^*∗∗*^	<0.0001^*∗∗∗*^	<0.0001^*∗∗∗*^	0.001^*∗∗*^	ns (0.050)	—	0.0004^*∗∗∗*^	0.0003^*∗∗∗*^
*p* value: injury versus no injury	0.011^*∗*^	0.004^*∗∗*^	0.0002^*∗∗∗*^	0.018^*∗*^	0.015^*∗*^	—	0.043^*∗*^	ns (0.078)

Around day 10 of life								
Healthy newborns	1.061 ± 0.017 (*n* = 4)	1.098 ± 0.022 (*n* = 4)	1.152 ± 0.014 (*n* = 4)	1.671 ± 0.111 (*n* = 4)	1.480 ± 0.077 (*n* = 4)	1.171 ± 0.037 (*n* = 4)	1.118 ± 0.064 (*n* = 4)	1.116 ± 0.043 (*n* = 4)
Asphyxiated newborns—no injury	1.090 ± 0.055 (*n* = 34)	1.102 ± 0.051 (*n* = 37)	1.170 ± 0.062 (*n* = 43)	1.675 ± 0.118 (*n* = 47)	1.678 ± 0.121 (*n* = 49)	1.171 ± 0.061 (*n* = 51)	1.123 ± 0.049 (*n* = 45)	1.133 ± 0.068 (*n* = 51)
Asphyxiated newborns—injury	1.154 ± 0.111 (*n* = 20)	1.120 ± 0.105 (*n* = 17)	1.201 ± 0.119 (*n* = 11)	1.725 ± 0.163 (*n* = 7)	1.622 ± 0.082 (*n* = 5)	1.211 ± 0.044 (*n* = 3)	1.124 ± 0.051 (*n* = 9)	1.132 ± 0.052 (*n* = 3)
*p* value: no injury versus healthy	ns	ns	ns	ns	0.0004^*∗∗∗*^	ns	ns	ns
*p* value: injury versus healthy	0.020^*∗*^	ns	ns	ns	0.0002^*∗∗∗*^	0.001^*∗∗∗*^	ns	ns
*p* value: injury versus no injury	0.009^*∗∗*^	ns	ns	ns	ns	0.015^*∗*^	ns	ns

Around day 30 of life								
Healthy newborns	1.020 ± 0.033 (*n* = 4)	1.040 ± 0.043 (*n* = 4)	1.086 ± 0.022 (*n* = 4)	1.508 ± 0.069 (*n* = 4)	1.379 ± 0.041 (*n* = 4)	1.082 ± 0.039 (*n* = 4)	1.055 ± 0.055 (*n* = 4)	1.113 ± 0.078 (*n* = 4)
Asphyxiated newborns—no injury	1.029 ± 0.045 (*n* = 14)	1.052 ± 0.044 (*n* = 16)	1.110 ± 0.056 (*n* = 21)	1.607 ± 0.182 (*n* = 22)	1.609 ± 0.179 (*n* = 23)	1.141 ± 0.062 (*n* = 26)	1.100 ± 0.051 (*n* = 23)	1.112 ± 0.063 (*n* = 25)
Asphyxiated newborns—injury	1.040 ± 0.052 (*n* = 13)	1.068 ± 0.044 (*n* = 11)	1.110 ± 0.062 (*n* = 6)	1.682 ± 0.207 (*n* = 5)	1.618 ± 0.110 (*n* = 4)	1.136 ± 0.058 (*n* = 1)	1.120 ± 0.050 (*n* = 4)	1.139 ± 0.056 (*n* = 2)
*p* value: no injury versus healthy	ns	ns	ns	ns	0.022^*∗*^	0.009^*∗∗*^	ns (0.052)	ns
*p* value: injury versus healthy	ns	ns	ns	0.043^*∗*^	<0.0001^*∗∗∗*^	—	0.026^*∗*^	—
*p* value: injury versus no injury	ns	ns	ns	ns	ns	—	ns	—

Mean ± standard deviation.

^*∗*^ *p* < 0.05, ^*∗∗*^ *p* < 0.005, and ^*∗∗∗*^ *p* < 0.0005.

**Table 3:** Comparison of fractional anisotropy (FA) values in each region of interest between healthy newborns and asphyxiated newborns treated with hypothermia developing or not developing brain injury, on day 1 of life, on days 2-3 of life, around day 10 of life, and around day 30 of life.

	Thalamus	Posterior limb of internal capsule	Lentiform nucleus	Anterior white matter	Posterior white matter	Frontal cortical grey matter	Parietal cortical grey matter	Occipital cortical grey matter
On day 1 of life								
Healthy newborns	0.167 ± 0.006 (*n* = 4)	0.524 ± 0.044 (*n* = 4)	0.136 ± 0.018 (*n* = 4)	0.134 ± 0.031 (*n* = 4)	0.176 ± 0.070 (*n* = 4)	0.153 ± 0.030 (*n* = 4)	0.148 ± 0.022 (*n* = 4)	0.154 ± 0.027 (*n* = 4)
Asphyxiated newborns—no injury	0.169 ± 0.016 (*n* = 6)	0.516 ± 0.049 (*n* = 6)	0.143 ± 0.017 (*n* = 8)	0.113 ± 0.027 (*n* = 12)	0.160 ± 0.035 (*n* = 12)	0.138 ± 0.018 (*n* = 13)	0.139 ± 0.018 (*n* = 8)	0.129 ± 0.031 (*n* = 13)
Asphyxiated newborns—injury	0.174 ± 0.018 (*n* = 7)	0.484 ± 0.031 (*n* = 7)	0.139 ± 0.012 (*n* = 5)	0.101 ± 0.027 (*n* = 1)	0.148 ± 0.020 (*n* = 1)		0.147 ± 0.019 (*n* = 5)	
*p* value: no injury versus healthy	ns	ns	ns	ns	ns	ns	ns	ns
*p* value: injury versus healthy	ns	ns	ns	—	—	—	ns	—
*p* value: injury versus no injury	ns	ns	ns	—	—	—	ns	—

On days 2-3 of life								
Healthy newborns	0.173 ± 0.012 (*n* = 4)	0.491 ± 0.033 (*n* = 4)	0.143 ± 0.026 (*n* = 4)	0.130 ± 0.018 (*n* = 4)	0.176 ± 0.028 (*n* = 4)	0.162 ± 0.017 (*n* = 4)	0.141 ± 0.038 (*n* = 4)	0.149 ± 0.034 (*n* = 4)
Asphyxiated newborns—no injury	0.165 ± 0.019 (*n* = 15)	0.484 ± 0.038 (*n* = 17)	0.142 ± 0.026 (*n* = 20)	0.128 ± 0.028 (*n* = 29)	0.154 ± 0.025 (*n* = 27)	0.150 ± 0.026 (*n* = 31)	0.146 ± 0.019 (*n* = 21)	0.136 ± 0.029 (*n* = 29)
Asphyxiated newborns—injury	0.173 ± 0.021 (*n* = 17)	0.478 ± 0.041 (*n* = 15)	0.142 ± 0.031 (*n* = 12)	0.128 ± 0.037 (*n* = 3)	0.162 ± 0.035 (*n* = 5)	0.165 ± 0.020 (*n* = 1)	0.139 ± 0.020 (*n* = 11)	0.167 ± 0.029 (*n* = 3)
*p* value: no injury versus healthy	ns	ns	ns	ns	ns	ns	ns	ns
*p* value: injury versus healthy	ns	ns	ns	ns	ns	—	ns	ns
*p* value: injury versus no injury	ns	ns	ns	ns	ns	—	ns	ns

Around day 10 of life								
Healthy newborns	0.175 ± 0.011 (*n* = 4)	0.503 ± 0.039 (*n* = 4)	0.156 ± 0.015 (*n* = 4)	0.135 ± 0.017 (*n* = 4)	0.186 ± 0.019 (*n* = 4)	0.159 ± 0.018 (*n* = 4)	0.127 ± 0.020 (*n* = 4)	0.133 ± 0.013 (*n* = 4)
Asphyxiated newborns—no injury	0.171 ± 0.020 (*n* = 34)	0.502 ± 0.040 (*n* = 37)	0.136 ± 0.019 (*n* = 43)	0.131 ± 0.026 (*n* = 47)	0.142 ± 0.031 (*n* = 49)	0.154 ± 0.037 (*n* = 51)	0.140 ± 0.026 (*n* = 45)	0.121 ± 0.026 (*n* = 51)
Asphyxiated newborns—injury	0.153 ± 0.027 (*n* = 20)	0.414 ± 0.040 (*n* = 17)	0.119 ± 0.014 (*n* = 11)	0.112 ± 0.027 (*n* = 7)	0.141 ± 0.025 (*n* = 5)	0.170 ± 0.066 (*n* = 3)	0.145 ± 0.027 (*n* = 9)	0.121 ± 0.020 (*n* = 3)
*p* value: no injury versus healthy	ns	ns	0.009^*∗∗*^	ns	0.001^*∗∗*^	ns	ns	ns
*p* value: injury versus healthy	0.048^*∗*^	0.0002^*∗∗∗*^	0.001^*∗∗*^	ns (0.087)	0.0003^*∗∗∗*^	ns	ns	ns
*p* value: injury versus no injury	0.021^*∗*^	<0.0001^*∗∗∗*^	0.0004^*∗∗∗*^	0.034^*∗*^	ns	ns	ns	ns

Around day 30 of life								
Healthy newborns	0.197 ± 0.014 (*n* = 4)	0.545 ± 0.028 (*n* = 4)	0.159 ± 0.012 (*n* = 4)	0.160 ± 0.016 (*n* = 4)	0.229 ± 0.042 (*n* = 4)	0.182 ± 0.047 (*n* = 4)	0.169 ± 0.027 (*n* = 4)	0.132 ± 0.019 (*n* = 4)
Asphyxiated newborns—no injury	0.195 ± 0.024 (*n* = 14)	0.543 ± 0.047 (*n* = 16)	0.154 ± 0.022 (*n* = 21)	0.142 ± 0.028 (*n* = 22)	0.186 ± 0.052 (*n* = 23)	0.154 ± 0.026 (*n* = 26)	0.160 ± 0.027 (*n* = 23)	0.139 ± 0.040 (*n* = 25)
Asphyxiated newborns—injury	0.173 ± 0.017 (*n* = 13)	0.474 ± 0.040 (*n* = 11)	0.145 ± 0.015 (*n* = 6)	0.115 ± 0.027 (*n* = 5)	0.153 ± 0.042 (*n* = 4)	0.180 ± 0.041 (*n* = 1)	0.173 ± 0.014 (*n* = 4)	0.169 ± 0.049 (*n* = 2)
*p* value: no injury versus healthy	ns	ns	ns	ns (0.074)	0.025^*∗*^	ns	ns	ns
*p* value: injury versus healthy	0.003^*∗∗*^	0.0001^*∗∗∗*^	0.022^*∗*^	0.004^*∗∗*^	0.0006^*∗∗∗*^	—	ns	—
*p* value: injury versus no injury	0.002^*∗∗*^	<0.0001^*∗∗∗*^	ns	0.011^*∗*^	ns	—	ns	—

Mean ± standard deviation

Injury versus no injury: ^*∗*^ *p* < 0.05, ^*∗∗*^ *p* < 0.005, and ^*∗∗∗*^ *p* < 0.0005.

## Abbreviations

ADC: Apparent diffusion coefficient
DTI: Diffusion-tensor imaging
DWI: Diffusion-weighted imaging
FA: Fractional anisotropy
MRI: Magnetic resonance imaging.
